# Through the Gerontological Looking Glass: Perspectives From Later Life

**DOI:** 10.1093/geroni/igaa057.2325

**Published:** 2020-12-16

**Authors:** Edward Ansello

**Affiliations:** Virginia Commonwealth University, Richmond, Virginia, United States

## Abstract

Do dominant gerontological research and education models help us understand our own aging? “(These) seemingly made no impact on my expectations for my own late life” (Cohen, 2017). The gerontological looking glass tends to favor research and teaching that identify large patterns, populations, and research cell sizes of “sufficient” numbers to produce data about external, descriptive assessments; and are compliant with nomothetic ideology: general, universal, and consistent. Even qualitative data rely on numbers of respondents to identify themes. In contrast, idiographic ideology emphasizes the individual and unique. While not meant to yield general findings, neither does it produce the incompleteness of descriptive statistical approaches, i.e., trying to capture the external persona. As human aging is both inherently universal and profoundly individual, one is likely to appreciate idiographic gerontology with awareness of one’s own aging. Various complementary perspectives—including humanistic gerontology, positive and conscious aging, and the curriculum palette—are relevant.

